# Profiling the injuries of law enforcement recruits during academy training: a retrospective cohort study

**DOI:** 10.1186/s13102-022-00533-y

**Published:** 2022-07-20

**Authors:** Danny J. Maupin, Elisa F. D. Canetti, Ben Schram, Robert G. Lockie, J. Jay Dawes, Joseph M. Dulla, Rob M. Orr

**Affiliations:** 1 Faculty of Health Sciences and Medicine, Bond University, 2 Promethean Way, Robina, QLD 4226 Australia; 2 Tactical Research Unit, Bond University, Robina, QLD 4226 Australia; 3 Department of Kinesiology, California State University, Fullerton, CA 92831 USA; 4 School of Kinesiology, Applied Health and Recreation, Oklahoma State University, Stillwater, OK 74078 USA; 5 OSU Tactical Fitness and Nutrition Lab, Oklahoma State University, Stillwater, OK 74078 USA

**Keywords:** Police, Fitness, Strength and conditioning, Needs analysis, Tactical

## Abstract

**Background:**

Injuries within law enforcement are a significant issue as they increase organisational costs and workforce strain. As one of the biggest risk factors of future injury is previous injury, minimising injuries suffered during academy has multiple beneficial and long-term effects, including a healthier and fitter police force. The purpose of this study was to profile the injuries sustained at a law enforcement academy to inform future injury mitigation strategies.

**Methods:**

Injury data were provided retrospectively (from May 2012 to September 2019) from the official insurance records of a law enforcement academy and included nature, location, and activity performed at time of injury. A total of 4340 (3288 males, 938 females, 114 sex not stated) recruits participated in academy training during this period. Inclusion criteria for the data were (a) injury record related to a recruit, and (b) the recruit was injured during academy training., with injury defined as tissue damage caused by acute or repetitive trauma, inclusive of musculoskeletal, neural, and/or integumentary systems but excluding general medical conditions such as cardiac (e.g. heart attacks) or respiratory (asthma) that was subsequently reported for worker’s compensation. Injury incidence rates and proportions were calculated and a Spearman’s correlation analysis was conducted between injury rates over successive classes.

**Results:**

An injury incidence rate of 368.63 injuries per 1000 recruits per year was calculated in this population, with a moderate correlation coefficient (r_s_ = 0.60) of increasing injury rates over chronologically occurring classes. Females also had higher injury rates than male recruits, even across various fitness levels. Trauma to joints and ligaments (49.30%) was the most common injury, and the knee the most common location (23.17%) of injury. Physical training (56.10%) was the most common activity being performed at the time of injury.

**Conclusions:**

This research demonstrates a large number of lower limb, musculoskeletal injuries that often occur during physical training. Further research is needed to assess suitable injury mitigation programs.

## Background

Law enforcement officers commonly encounter physically stressful situations [1], such as foot pursuits and restraining uncooperative suspects [2]. The physical nature of law enforcement increases the risk of officers suffering musculoskeletal injuries, such as muscle strains [3]. It has been reported that law enforcement officers suffer musculoskeletal injuries at a rate ranging from 240 to 2500 per 1000 personnel per annum [3]. The high injury rates seen in law enforcement officers has a combination of detrimental side effects both at an individual and organisational level.

Injuries have the potential to increase an officer’s risk of future injury and decreasing their occupational performance, as referenced in military populations [4–6]. This is of particular concern as previous investigations have reported that having sustained a previous injury is the strongest predictor of future injury [5]. A systematic review across active populations, including military, found that previous lower extremity injuries significantly increased the risk of future injury due to a variety of potential factors, such decreased strength, proprioception, and neuromuscular control [5]. These findings highlight the importance of interventions to reduce early-career injuries. Two systematic reviews report a similar injury profile between law enforcement recruits and current officers, with a majority of injuries occurring in the upper limb and spine [3, 7]. It does appear that law enforcement recruits may have a larger proportion of lower extremity injuries, though the overall quality of included studies was remarked to below quality [7]. Preventing injuries during this time period may have a significant impact on future injuries as a previous injury history has been identified as a significant risk factor for future injury [8]. A possible strategy to mitigate injury rates is to target injury prevention programs to law enforcement recruits while they are completing mandatory academy training.

During academy training, physical training periods are typically implemented to develop the fitness and resilience required to perform physically demanding occupational tasks [9]. Law enforcement recruits are typically drawn from the general population and often have varying levels of physical training experience and fitness prior to the commencement of training which may influence the intensity of training an individual can tolerate [1]. A decreased ability to tolerate intense physical activity may be a contributing factor to the increased risk of injury in recruits with lower self-rated physical activity and frequency of exercise [8]. The transition from general life to the highly physical, mental, and emotional demands of academy training can result in a drastic increase in a recruit’s overall level of stress, potentially leading to increased risk of injury [10]. In military personnel, this increase in stress (inclusive of physical and mental stress) is potentially one of reasons recruits commonly experience a higher rate of injury than those active duty [11]. Though different professions, with unique occupational demands, both law enforcement and military utilise training academies with similar aims to improve physical fitness and knowledge [9, 11]. While the training academies have differing demands (e.g., military recruits tend to sleep on base, while law enforcement recruits tend to return home), both academies result significant increases in physical and mental stress [12–14]. It is conceivable that law enforcement recruits entering a similarly demanding training period may also be experiencing increased risk of injury. While law enforcement is an inherently unpredictable profession, academy training tends to be more controlled in nature, where academy staff are better able to manage risk exposure [15].

The controlled nature of the training environment presents a unique opportunity to implement injury mitigation strategies that may reduce a recruit’s injury potential. Previous strategies, including ability based training [16] and periodised strength training and conditioning [17], have been hypothesised as being able to reduce injuries. To further optimise effectiveness of injury mitigation programs it is imperative that are specific and targeted to a given population [11]. This is due in part to differing physical training programs between agencies [18] and varying fitness levels of recruits between agencies even within the same country [19]. For this reason, a detailed injury analysis within the population is required to develop appropriate intervention strategies.

Injury profiles provide further additional benefits, such as time lost and financial cost [20]. These factors are especially crucial in law enforcement as any time lost will require officers to complete additional shifts thus increasing their risk of injury through additional workplace exposure. Previous research has suggested that approximately 26% of officers can miss 30 or more days due to injury [21]. Injuries also incur a financial cost to treat and rehabilitate, ranging from $2,500 to $12,000 USD [22]. Building specific injury profiles improve the understanding of a specific organisations time and financial costs of injuries, as well as aid in designing effective programs to mitigate these negative consequences.

Developing injury profiles, consisting of common injuries and their mechanisms, specific to a given academy allows for more targeted injury mitigation strategies to be developed and employed. In addition, they can allow for further information on time lost and financial costings of injuries. As such, the aim of this study was to profile the injuries sustained by recruits during academy training from one specific United States (US) based law enforcement academy to inform future injury mitigation strategies and to develop a baseline against which future mitigation strategies could be measured.

## Methods

### Participants

This retrospective study reviewed recruit injury and illness data recorded by a US law enforcement academy from May 2012 to September 2019. During this time period, a total of 4340 (3288 males, 938 females, 114 sex not stated) recruits participated in academy training, across 52 classes. Further demographic data was not provided, a common finding in tactical populations due, in part, to security concerns [1, 16, 23]. Records for academy recruits were drawn from the academy’s worker’s compensation insurance database and consisted of the official records of academy recruit members injured. This study received ethics approval from Bond University and California State University, Fullerton ethics committees under HSR-17-0037. Due to the retrospective nature of this study, a waiver of consent was sought and granted. Permission to use the data was also obtained from the law enforcement academy from which the data were drawn.

### Procedures

De-identified data included injury date, claim number, body site, nature of the injury, activity performed at time of injury, position in the academy, age, a narrative description of the incident, as well as the appropriate codes, using the International Classification of Disease (ICD-9 or ICD-10) used to describe these injuries. This coding was originally performed by the diagnosing physician. During this time frame, the police organization noted that the length of academy training varied from 20 to 22 weeks. Injury for this study was defined as tissue damage caused by acute or repetitive trauma, inclusive of musculoskeletal, neural, and/or integumentary systems but excluding general medical conditions such as cardiac (e.g. heart attacks) or respiratory (asthma) that was subsequently reported for worker’s compensation. It must be noted that not all injuries resulted in time off from work. In this population group, all reported injuries are subsequently processed under insurance claims, thus the filed insurance claims are an accurate representation of reported injuries. Fitness data was also included to allow for further analysis on the relationship between fitness levels and injuries. Fitness was measured via a standardised testing battery (the PT500), which has been described previously in multiple research papers [9, 24–26], and split into quintiles.

Inclusion criteria for the data were (a) injury record related to a recruit, and (b) the recruit was injured during academy training. Records were excluded if (a) the data were incomplete and thus not being able to identify the nature or location of the injury, (b) the data were a duplicate entry, or (c) the claim was in relation to illness. The data were manually reviewed to ensure only eligible records were included for analysis. Non-eligible and duplicate records were removed using the criteria previously described. If one recruit exhibited multiple ICD codes for the same location under the same claim, only the injury that most closely reflected the overall diagnosis or the most severe injury were counted towards the total. For example, if it was acknowledged that the recruit had an ankle joint and ligament injury in addition to an ankle fracture, only the data associated with the ankle fracture were utilised. Bilateral injuries were counted as two distinct entries.

### Data analysis

Data were reclassified according to ICD-10 codes, if not already present, to allow easier grouping of nature of injuries for presentation. This was completed by utilising the ICD codes originally applied, as well as the free-text narrative. Where inconsistencies existed between the free-text narrative and ICD codes, precedence was given to the narrative as this can present a more detailed overview compared to a finite coding system [27]. This process was performed by the lead author, a qualified physiotherapist with assistance from a second author (BS) who has previously completed a similar strategy and is also a qualified physiotherapist. An overview of this process can be seen in Appendix 1. ICD-10 codes were further aggregated into overall categories to allow for more efficient and clearer presentation of data. For example, a sprain of joint or body area was classified as trauma to joint/ligaments, while tendinopathy and strain of muscle, among others, were classified as injury to muscle/tendon.

Recruits were placed into quintiles based on initial fitness testing scores, which was referred to as the PT500. Initial fitness scores were only provided for a subset of the population (15 classes, 1133 recruits, 219 females, 914 males). IRs were calculated per quintile. The potential for recruits to be dismissed from the academy was factored into the count of weeks of training completed. If a recruit was dismissed, the week of the dismissal was counted towards the week totals with the remainder of weeks left unaccounted. For clarity, all weeks in which recruits were exposed to training were counted. For those recruits who successfully completed training, this was 20 or 22 weeks (depending on their respective course length) of training. For those recruits who were separated before the end of training (dropped out), all weeks of training up to and including the week in which they separated were included, as they were exposed to training in those weeks. Later weeks, in which these latter recruits were *not* continuing with training and so *not* exposed to training were not counted and so did not contribute towards the total cohort exposure time. This meant that only valid weeks of exposure to training were counted in the denominator when calculating incidence rates for injuries that occurred during training. However, details of separations were unavailable for one class, with no information provided. For the class where the details of separations were unavailable an estimate was calculated by taking the average of weeks (rounded to the nearest whole week, resulting in an average of 240 weeks) of separation of all classes with the same course length and applied at a cohort level. To further improve the accuracy of IRs, only injuries that occurred during completed classes were counted. Injuries that occurred in classes that had started prior to May 2012 or ended after September 2019 were excluded to ensure consistency.

### Statistical analysis

Following the data cleaning process, incidence rates (IR) per 1000 recruits per year of exposure to training were calculated. This was achieved by firstly determining the number of injuries per recruit per week, calculated by dividing the total number of injuries observed by the total number of weeks recruits were exposed to training. Next, this result was multiplied by the number of weeks in a year (i.e. 52) to calculate the number of injuries per recruit per year, and then by 1000 to determine the number of injuries per 1000 recruits per year of exposure to training, proving an overall exposure to training for all recruits. Conversion to number of injuries per 1000 recruits per year of exposure to training allows for further comparison across multiple tactical and police populations regardless of training duration. A 95% confidence interval was determined using the online calculator at https://www.openepi.com/PersonTime1/PersonTime1.htm and utilising the Mid-P exact test [28]. This was performed for both male and female recruits.

The total number of injuries per individual class was then calculated and imported to R Studio (Version 1.2.5042, RStudio, Inc.) for correlation analysis to examine injury rates across successive (i.e., chronologically occurring) classes. Due to violations of normality as measured by Shapiro–Wilk test, Spearman’s rho was used to calculate correlations [29]. Spearman (r_s_) correlations were interpreted as follows: 0.00 as zero, 0.10–0.20 as poor, 0.30–0.50 as fair, 0.50–0.70 as moderate, 0.80–0.90 as very strong, 1.00 as perfect [30]. Of note, the correlation analysis was only used to assess the correlation between incidence rates and passage of time as consecutive classes completed training. Next the nature of injury, location of injury, and activity performed at time of injury are presented as a raw figure and percentage of total amount of injuries. These figures were then tabulated to identify the most common nature and location of injury in this population as well as the activity being performed when injury occurred. The 95% confidence intervals were calculated for the injury, location of injury, and activity performed at time of injury proportions. This was completed using an online calculator found here: http://vassarstats.net/prop1.html.

Incidence rate ratios (IRR) were subsequently calculated to provide further comparisons of injury rate across fitness quintiles (using the lowest fitness quintile as the reference group), time, and sex. Determining the IRR for time was completed by dividing the IR during the first five chronological classes by the IR for the last five chronological classes. The 95% confidence intervals around the IRR were calculated as follows [31]:

$${95}\% {\text{CI}} = {\text{exp }}\left( {{\text{ln }}\left[ {{\text{IRR}}} \right] - {1}.{96} \times {\text{SE}}\left( {{\text{ln}}\left[ {{\text{IRR}}} \right]} \right)} \right){\text{ to exp }}\left( {{\text{ln}}\left[ {{\text{IRR}}} \right] + {1}.{96} \times {\text{SE}}\left( {{\text{ln}}\left[ {{\text{IRR}}} \right]} \right)} \right)$$

where

$${\text{SE}}\left( {{\text{ ln}}\left[ {{\text{IRR}}} \right]} \right) = \surd \left( {{1}/\left[ {{\text{IR}}_{{\text{A}}} } \right] + {1}/\left[ {{\text{IR}}_{{\text{B}}} } \right]{-}{1}/{\text{IR}}_{{\text{B}}} {-}{1}/{\text{IR}}_{{\text{A}}} } \right)$$

## Results

A total of 568 (194 females, 366 males, 8 unknown) injuries were identified during this seven-year period. This resulted in an IR of 376.51 (95% CI 345.50–409.60) injuries per 1000 recruits per year of exposure. Females had an IR of 650.97 (95% CI 560.00–752.60) injuries per 1000 recruits per year of exposure, while males had an incidence of 311.37(95% CI 279.80–345.50) injuries per 1000 recruits per year of exposure. The calculated IRR between male and female IR was 2.09 (95% CI 1.85–2.37).

### Injury characteristics

The most common nature of injury that occurred was trauma to joints and ligaments (49.47% of injuries), followed by injury to muscle (26.41%), and fractures (7.21%) (Table 1). Regarding the location of injury, the most common area injured was the knee (23.42%) followed by the ankle (13.91%) and lower leg (10.39%) (Table 2). The five most common injured areas all concern the lower extremity and are then followed by the low back (6.34%) and shoulder (5.46%).

**Table 1:** Nature of reported injuries

Nature	Proportion of injuries (%)	95% CI	Incidence	Proportion of injuries—male (%)	95% CI	Proportion of injuries—female (%)	95% CI
Trauma to joints/ligaments	281 (49.47%)	45.38–53.57%	181.50	176 (48.09%)	43.02–53.20%	101 (52.06%)	45.06–58.98%
Injury to Muscle	150 (26.41%)	22.95–30.19%	98.77	97 (26.50%)	22.24–31.25%	51 (26.29%)	20.60–32.90%
Fracture	41 (7.21%)	5.37–9.65%	26.30	27 (7.33%)	5.12–10.52%	13 (6.70%)	3.96–11.12%
Contusion	38 (6.69%)	4.91–9.05%	24.37	26 (7.10%	4.89–10.20%	12 (6.19%)	3.58–10.50%
Superficial Injury	31 (5.46%)	3.87–7.65%	19.88	22 (6.01%)	4.00–8.93%	8 (4.12%)	2.10–7.92%
Dislocation	12 (2.11%)	1.21–3.65%	7.70	7 (1.91%)	0.93–3.89%	5 (2.58%)	1.11–5.89%
Concussion	10 (1.76%)	0.96–3.21%	6.41	7 (1.91%)	0.93–3.89%	3 (1.55%)	0.53–4.45%
Injury to Nerve	5 (0.88%)	0.48–2.04%	3.21	4 (1.09%)	0.42–2.77%	1 (0.52%)	0.09–2.87%
Total	568 (100%)			366 (100%)		194 (100%)	

Key: Incidence reported in cases per 1000 recruits per year of exposure to training. Columns may not add up to 100% due to rounding

**Table 2:** Body location of reported injuries

Location	Proportion of injuries (%)	95% CI	Incidence	Proportion of injuries—male (%)	95% CI	Proportion of injuries—female (%)	95% CI
Knee	133 (23.42%)	20.12–27.07%	85.30	88 (24.04%)	19.95–28.67%	42 (21.65%)	16.44–27.97%
Ankle	79 (13.91%)	11.31–17.00%	50.67	50 (13.66%)	10.52–17.56%	29 (14.85%)	10.62–20.65%
Lower Leg	59 (10.39%)	8.14–13.17%	40.41	37 (10.11%)	7.42–13.62%	20 (10.31%)	6.77–15.39%
Hips	50 (8.80%)	6.74–11.42%	32.07	27 (7.38%)	5.12–10.52%	22 (11.34%)	7.61–16.57%
Thigh	40 (7.04%)	5.21–9.45%	25.65	30 (8.20%)	5.80–11.46%	10 (5.16%)	2.82–9.22%
Low Back	36 (6.34%)	4.61–8.65%	23.09	27 (7.38%)	5.12–10.52%	9 (4.64%)	2.46–8.58%
Shoulder	32 (5.63%)	4.02–7.84%	21.16	20 (5.47%)	3.56–8.28%	12 (6.19%)	3.58–10.50%
Foot/Toe	26 (4.58%)	3.14–6.63%	16.68	15 (4.10%)	2.50–6.65%	11 (5.67%)	3.20–9.87%
Hand/Digit	24 (4.22%)	2.86–6.22%	15.39	15 (4.10%)	2.50–6.65%	9 (4.64%)	2.46–8.58%
Face	14 (2.46%)	1.47–4.09%	8.98	9 (2.50%)	1.30–4.61%	5 (2.58%)	1.11–5.89%
Chest/Rib	13 (2.29%)	1.34–3.88%	8.34	7 (1.91%)	0.93–3.89%	5 (2.58%)	1.11–5.89%
Nervous System	11 (1.94%)	1.09–3.44%	7.05	8 (2.19%)	1.11–4.26%	3 (1.55%)	0.53–4.45%
Head	10 (1.76%)	0.96–3.21%	6.41	8 (2.19%)	1.11–4.26%	2 (1.03%)	0.28–3.68%
Wrist	9 (1.59%)	0.83–2.98%	6.41	3 (0.82%)	0.28–2.38%	6 (3.09%)	1.42–6.58%
Ears	6 (1.06%)	0.49–2.29%	3.85	2 (0.55%)	0.15–1.98%	4 (2.06%)	0.80–5.18%
Neck	6 (1.06%)	0.49–2.29%	3.85	5 (1.37%)	0.59–3.16%	1 (0.52%)	0.09–2.87%
Eyes	5 (0.88%)	0.38–2.04%	3.21	4 (1.09%)	0.42–2.77%	0	0
Abdomen	5 (0.88%)	0.38–2.04%	3.21	2 (0.55%)	0.15–1.98%	3 (1.54%)	0.53–4.45%
Mouth/Throat	4 (0.70%)	0.27–1.79%	2.57	4 (1.09%)	0.42–2.77%	0	0
Upper Arm	3 (0.53%)	0.18–1.65%	1.92	3 (0.82%)	0.28–2.38%	0	0
Elbow	2 (0.35%)	0.10–1.27%	1.28	1 (0.27%)	0.05–1.53%	1 (0.52%)	0.09–2.87%
Upper Back	1 (0.18%)	0.03–1.00%	0.64	1 (0.27%)	0.05–1.53%	0	0
Total	568 (100%)			366 (100%)		194 (100%)	

(1) Incidence reported in cases per 1000 recruits per year of exposure to training. (2) Nervous system is inclusive of concussions. Columns may not add up to 100% due to rounding

Over half (55.63%) of all injuries experienced over the course of this seven-year period occurred during physical training, while 19.89% of injuries occurred during defensive tactics training, where recruits learn how to defend themselves from aggressive and violent suspects (Table 3). Approximately 8.98% of injuries occurred during an unknown activity, though it appears these injuries were commonly musculoskeletal in nature and a combination of overuse and acute injuries.

**Table 3:** Activity performed when injury occurred

Activity	Proportion of injuries (%)	95% CI	Incidence	Proportion of injuries—male (%)	95% CI	Proportion of injuries—female (%)	95% CI
Physical Training	316 (55.63%)	51.52–59.66%	206.51	199 (54.37%)	49.25–59.40%	115 (59.28%)	52.25–65.95%
Defensive Tactics	113 (19.89%)	16.81–23.37%	72.47	87 (23.77%)	19.70–28.39%	24 (12.37%)	8.46–17.75%
Unknown	51 (8.98%)	6.90–11.61%	32.71	29 (7.92%)	5.57–11.14%	20 (10.31%)	6.77–15.39%
Occupational Simulations	42 (7.39%)	5.51–9.84%	26.94	26 (7.10%)	4.89–10.20%	14 (7.22%)	4.35–11.75%
Marching	12 (2.11)%	1.21–3.65%	7.70	9 (2.46%)	1.30–4.61%	13 (6.70%)	3.96–11.12%
Range	8 (1.41%)	0.72–2.76%	5.13	3 (0.82%)	0.28–2.38%	5 (2.58%)	1.11–5.89%
Personal Hygiene	7 (1.23%)	0.60–2.52%	4.49	5 (1.36%)	0.59–3.16%	2 (1.03%)	0.28–3.68%
Manual Handling	5 (0.88%)	0.38–2.04%	3.21	5 (1.36%)	0.59–3.16%	0	
Class	1 (0.18%)	0.03–1.00%	0.64	1 (0.27%)	0.05–1.53%	0	
Standing	1 (0.18%)	0.03–1.00%	0.64	1 (0.27%)	0.05–1.53%	0	
Total	568 (100%)			366 (100%)		194 (100%)	

(1) Incidence reported in cases per 1000 recruits per year of exposure of training. (2) Unknown injuries had insufficient data to classify activity at time of injury. Columns may not add up to 100% due to rounding

### Injuries rates across fitness levels and time

Fitness appeared to have an impact on injury incidence, both overall and between sexes, with lower quintiles having higher IRs (Table 4). IRs were highest in the 20th percentiles (IR: 891.57, 95% CI 723.80–1146.00 injuries per 1000 recruits per year of exposure) and decreased to the 100th percentiles (IR: 306.53, 95% CI 207.70–437.10 injuries per 1000 recruits per year of exposure). This is reflected in the relative IRR, decreasing from 0.42 (95% CI 0.32–0.55) in the 20th to 40th percentile comparison to 0.34 (95% CI 0.25–0.47) in the 20th to 100th percentile grouping. Male IRs followed a similar pattern with decreased rates across higher initial levels of fitness. The IRR follows a similar decrease across quintiles except for the 20th to 80th percentile comparison (IRR 0.46, 95% CI 0.31–0.68) being greater than the 20th to 60th percentile comparison (IRR 0.43, 95% CI 0.28–0.66). However, for females, while a lower IR was present in the 40th percentile (IR: 491.83, 95% CI 258.60–854.70 injuries per 1000 recruits per year of exposure), the IR increased across the 60th (IR: 587.20, 95% CI 286.30–1070.70), 80th (IR: 712.33, 95% CI 181.30–1939.00), and 100th (IR: 1162.01, 369.50–2805.00) percentiles. This resulted in increasing IRRs from the 20th to 40th percentile comparison (IRR: 0.42, 95% CI 0.32–0.55) to the 20th to 100th percentile comparison (IRR: 0.95, 95%CI 0.29–2.46).

**Table 4:** Incidence rates across fitness quintiles

	20th Percentile			40th Percentile			60th Percentile			80th Percentile			100th Percentile		
	Total (n = 229)	Female (n = 103)	Male (n = 126)	Total (n = 230)	Female (n = 58)	Male (n = 172)	Total (n = 224)	Female (n = 39)	Male (n = 184)	Total (n = 227)	Female (n = 10)	Male (n = 217)	Total (n = 224)	Female (n = 9)	Male (n = 215)
PT500 (mean ± SD)	181.25 ± 36.72	173.17 ± 38.72	187.86 ± 33.75	257.61 ± 16.54	256.48 ± 6.35	257.99 ± 16.63	311.58 ± 15.07	312.31 ± 14.28	311.42 ± 15.26	365.96 ± 17.11	361.90 ± 15.84	366.14 ± 17.18	439.20 ± 30.81	423.33 ± 6.41	439.86 ± 30.76
Injuries	71	42	29	36	11	25	29	9	20	29	3	26	28	4	24
IR (per 1000 recruits per year of exposure)	891.57	1212.66	644.44	407.05	491.83	378.35	328.83	587.20	274.48	314.43	712.33	295.39	306.53	1162.01	273.03
IR 95% CI (per 1000 recruits per year of exposure)	701.60 to 1118.00	885.10 to 1623.00	439.80 to 913.50	289.40 to 557.40	258.60 to 854.70	250.30 to 550.30	224.40 to 466.10	286.30 to 1077.00	172.40 to 416.40	214.60 to 445.70	181.30 to 1939.00	197.10 to 426.60	207.70 to 437.10	369.50 to 2805.00	179.00 to 400.10
IRR (per 1000 recruits per year of exposure)	1.00 (Ref)	1.00 (Ref)	1.00 (Ref)	0.42	0.41	0.59	0.37	0.48	0.43	0.35	0.59	0.46	0.34	0.95	0.42
95% CI IRR (per 1000 recruits per year of exposure)	–	–	–	0.32 to 0.55	0.27 to 0.60	0.40 to 0.86	0.27 to 0.50	0.33 to 0.70	0.28–0.66	0.26 to 0.48	0.33 to 1.06	0.31 to 0.68	0.25 to 0.47	0.29 to 2.46	0.28 to 0.63

*IR* Incidence Rate, 95% *CI* 95% Confidence Interval, *IRR* Incidence Rate Ratio; *Ref* Reference value

**Fig. 1:**
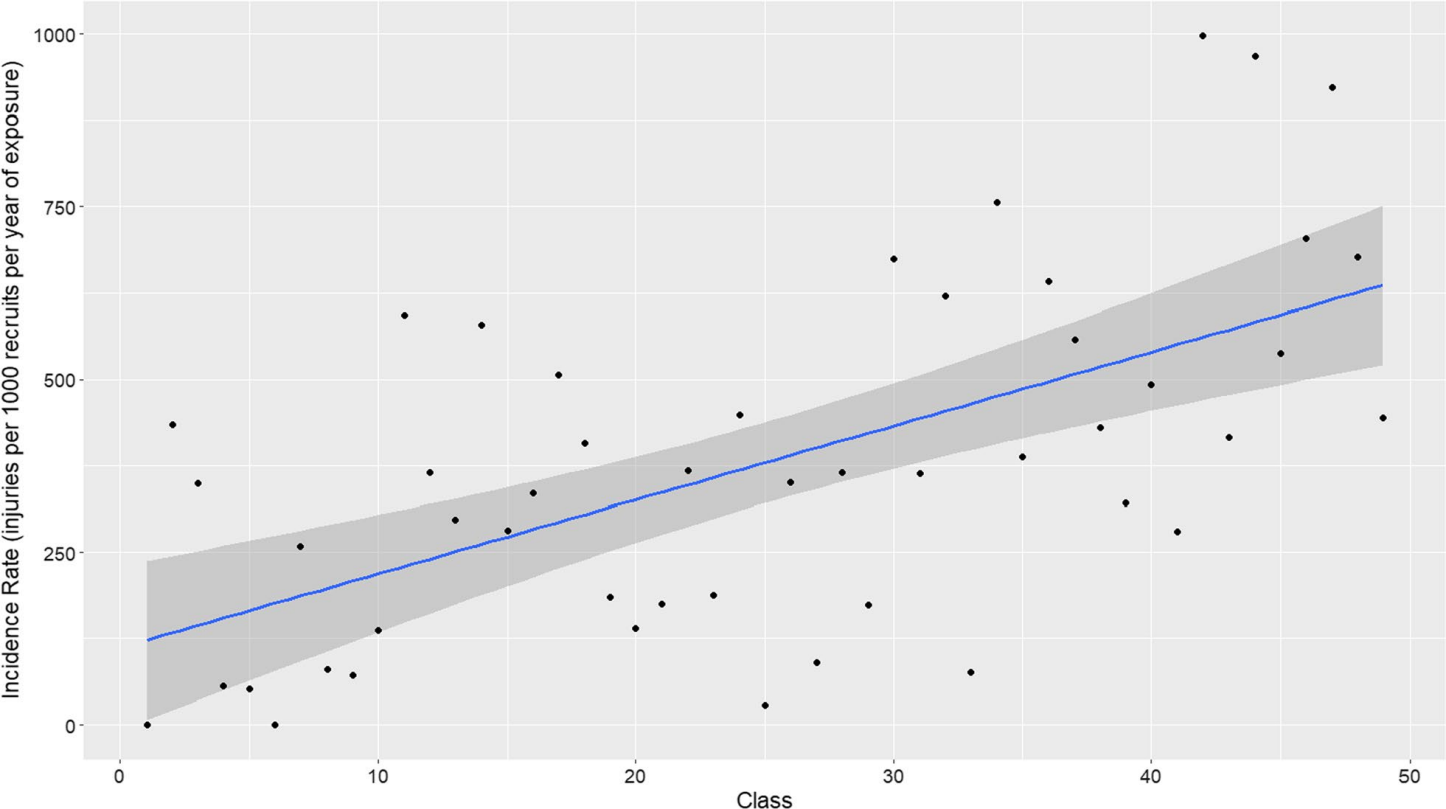
Injury rates per individual classes. Key: Each dot represents one recruit class, with classes ordered chronologically beginning in May 2012, with a line of best fit and 95% confidence interval

When plotting injuries across consecutive classes, there was a significant, positive trend for increased injury rates over successive classes with a moderate correlation coefficient (r_s_ = 0.60; *p*-value < 0.001) (Fig. 1). IRs were calculated for the first five (IR: 189.75, 95% CI 126.66–274.10 injuries per 1000 recruits per year of exposure) and last five (IR: 666.49 95% CI 544.60–807.90 injuries per 1000 recruits per year of exposure) classes (chronologically). A calculated IRR of 3.51 (95%CI 2.44 to 5.06) between these two groups, further suggest an increase in injuries over time.

**Fig. 2:**
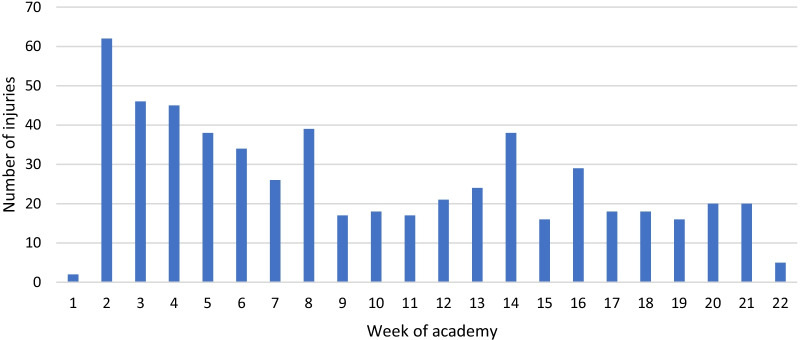
Number of injuries per week between during academy training from May 2012 to September 2019

The highest number of injuries occurred between weeks two and five, with the highest amount occurring during the second week (Fig. 2). Two other spikes occur during weeks eight and 14, with approximately 40 injuries occurring each week.

## Discussion

The aim of this study was to profile the injuries typically experienced by law enforcement recruits during academy training. This was undertaken to inform future injury mitigation strategies to potentially reduce injuries and their negative effects. Firstly, IRs in this study were found to be higher in females compared to males. This echoed previous research conducted by Schram et al. [32] in a tactical population. Schram et al. [32] also reported that injury rates between females and males were no longer significantly different after accounting for fitness levels. This is consistent with previous research in military populations that showed higher injury rates in females may be more likely due to differences in fitness levels rather than sex [33–36]. In contrast, a systematic review on injury rates in sports found male athletes to have a significantly higher injury rate [37]. While tactical and athletic populations have differing occupational demands this does provide further context that fitness levels in female tactical personnel may be impacting injury rates more than physiological or biomechanical differences [32]. Interestingly, female IR increased across higher fitness levels (although with overlap between confidence intervals), contrary to previous research which suggests higher fitness levels decrease risk of injury [1, 38]. The large differences between the 60th, 80th, and 100th percentiles, as well as the increase in injury across higher fitness level, may be due to the small female sample size in these groups. However, the previous studies that showed no significant differences when accounting for fitness had similar disparities in the sample populations [33–36]. Also of note, are the large 95% CI’s suggesting a wide range of IR’s over repeated samples and impacting the strength of findings. The studies by Tomes et al. [38] and Orr et al. [1] that linked fitness to injuries in law enforcement recruits did not contain information regarding the sex of participants so comparison of sample size, as well as fitness across sex, could not be made. Due to these potential limiting factors (e.g., sample size), it should not be accepted that females of with higher fitness levels will be at an increased risk of injury without further analysis. Future research could implement statistical analysis, such as appropriate linear regressions, with larger sample sizes to further assess the impact of fitness on injury risk.

In addition to the impact of fitness on injury rates, it has been reported that females may be more likely to report their injuries more frequently and sooner than male military recruits [32, 36], while in law enforcement it has been found that a higher proportion of female officers collected worker’s compensation [39]. Future research is needed to understand how this may impact reporting of injuries in law enforcement recruits between males and females [39]. These differences would suggest that the male injury rate would be underrepresented. Lastly, this study did not specifically account for fitness levels but rather investigated IRs within percentiles, which limits statistical findings and ability to control confounding variables. For example, females in the 60th percentile may still have significantly lower fitness levels than males within the same percentile which may influence injury rate. The decreasing sample size of female recruits in higher fitness quintiles may also impact the calculated IRs. As female recruits were found to have a higher IR in this population, a decrease in the proportion of female recruits in higher fitness quintiles may contribute to a smaller IR. Future research, utilising appropriate statistical analysis, will need to be conducted to further analyse the true effect of fitness on injury rates both between sexes within this population, and within females.

The findings from this study revealed that recruits at this academy were more likely to experience musculoskeletal injuries to their lower limb due to physical training or defensive tactics training. This is not entirely unexpected based on the physical demands and exertion associated with these forms of training, particularly physical training which typically includes body weight exercise and long distance running in this population [40, 41]. The nature of injuries in this populations is similar to previous research in a population of New Zealand recruits, consisting of mainly muscular and ligamentous injuries [42]. However, recruits in the New Zealand academy were more likely to suffer shoulder injuries which may reflect a difference in training methodology, though the research by Sawyer et al. [42] did not differentiate between physical training and defensive tactics. As defensive tactics focuses on training self-defence techniques, it is possible that more upper limb injuries may occur during this activity when compared to physical training. Future research should differentiate these two activities as a higher proportion of injuries during one activity may impact future mitigation strategies.

The high rate of upper limb injuries in New Zealand recruits is more closely related to the injury profile seen in law enforcement officers, as law enforcement officers commonly experience injuries to the upper limb [3]. This discrepancy between upper and lower limb injuries may be due to law enforcement officers engaging with and subduing suspects rather than long distance runs [3]. Although law enforcement officers are more likely to experience upper limb and back injuries [3], lower limb injuries can account for 13–30% of all injuries [43–45]. These injuries were found to typically be sprains and strains, and most often occurred at the knee and ankle [45]. As one of the best predictors of future injury is having suffered a previous injury, the high number of strains and sprains suffered by law enforcement recruits at this academy may influence future lower extremity injuries when working as officers. However, this would need to be confirmed with further prospective research studies.

While differing from other research in law enforcement populations [42, 45], the injury profile presented in this current study is similar to previous findings in military recruits. Injuries in military recruits are more likely to occur at or below the knee and consist of overuse or stress syndromes, muscle strains, and ankle sprains [46]. Though differences exist between military recruits and the population in the current study, both groups are usually drawn from the general population and focus on body weight exercises and long distance running for training [41, 47]. Research by Trank et al. [48], conducted in a military recruit population, has suggested that large amounts of running distance (> 25 mi or 40.23 km) leads to an increased risk of injury. A training program that focuses on long distance running may be predisposing recruits of this population to a large amount of lower limb injuries through mechanisms such as program-induced cumulative overload (PICO), whereby the combination of physical training and occupational demands can lead to injury [49]. Given the proportion of injuries occurring during physical training, future research into injury mitigation programs should examine the potential impacts of periodised physical training programs, ability-based training (where recruits training at level similar to their physical capacity) and upskilling of physical training instructors (through educational initiatives). The use of periodisation and education of academy staff may help to limit overtraining and contribute to a more gradual increase of physical loading in recruits. Future research is necessary to adequately explore, implement, and measure the effect of such intervention programs.

Data from this study also depicted a trend of increased injuries over successive classes (i.e., time) within this agency with greater IR during the end of the reporting session than the beginning, and no overlap between the calculated confidence intervals. The reason behind this increase in injuries over time is unknown. One potential explanation is increased sensitivity to, and better reporting of, injuries by academy staff, allowing them to report injuries that may have otherwise been missed. However, anecdotal information provided by training staff suggests that reporting standards did not change during this time period. Another possibility may be a general decline in the average fitness and an increase in obesity in recruits attending the training. This supposition is in-line with trends seen in general populations [50]. Lower fitness levels are associated with an increased risk of injuries [1, 38], with high body mass index also being a risk factor for injury in military recruits [51]. Previous research has shown that physical fitness levels, particularly aerobic fitness, muscular strength, and muscular power, are also predictive factors in recruit graduation [25, 52]. The general decline in public fitness levels may be having a negative effect on injury and graduation rate of law enforcement recruits. The academy in the present study does currently employ conditioning sessions prior to starting academy training, but these sessions are optional. Enforcing these sessions or supplying a physical training program for prospective recruits to begin prior to starting the academy may be a viable way of improving fitness and decreasing the risk of injuries prior to starting the academy.

While this study demonstrated a comprehensive injury profile of a US law enforcement agency, several limitations should be acknowledged. Firstly, studies that have employed a similar approach (such as using worker’s compensation data or similar reporting systems) have suggested the number of injuries may be underrepresented due to requiring a formal report [27, 53]. While both studies involved military populations, it is possible that a similar pattern exists with policing populations and this study may underrepresent injuries. As the injuries were taken from insurance claims through worker’s compensation, minor and less severe injuries may not have been reported, potentially leading to an underrepresentation of the incidence rates and influence the locations, natures and mechanisms of injuries sustained. Secondly, the free-text narrative was often incomplete with breaks occurring in the middle of a sentence. This resulted in a lack of information, which may have affected both the nature and activity being performed at time of injury. Another limitation is the use of one author to conduct the screening process of injury records, as this may increase the likelihood of errors and inappropriate exclusions when compared to utilising multiple authors. Information regarding financial cost and time loss was also not reported in this current study. Future research is necessary in this population to understand the extent of these organisational costs. As the aim of this article was provide a profile of injuries within this population, further statistical analysis (e.g., regression) was not conducted, deemed to be outside of the scope of this particular paper. Future analysis utilising these statistical tools would provide further evidence concerning injury risk with female recruits and those with lower levels of physical fitness. Lastly, the data reported here applies to one specific agency in the US and may not be representative of other law enforcement jurisdictions due to differing requirements and fitness levels. Though this allows for greater specificity in injury mitigation programs (such as periodisation, ability-based training, and upskilling of physical training instructors) for this agency, the findings of this study may not accurately reflect the injuries seen in other academies and should not be used in that regard.

## Conclusion

Law enforcement recruits from the US academy reported in this study suffered from a high rate of lower limb injuries, usually musculoskeletal in nature, and commonly caused by participation in physical training sessions. Further research in this population, and specific to each agency, should address potential strategies to minimise these injuries. Given the proportion of injuries occurring during physical training and lower limb, potential options to reduce injuries include ability-based training, upskilling of physical training instructors, and occupation-specific periodisation. Implementation and research of these mitigation strategies on injury risk in this population provide a future research and practical opportunity. Although similarities existed between the injury profiles of the recruits compared in this study, it is still necessary to continue to profile recruit injuries specific to each law enforcement agency to assist with the development of specific and targeted injury mitigation strategies.

## Data Availability

The datasets generated and/or analysed during this study are available from the corresponding author on reasonable request.

## Appendix 1

Overview of the injury screening process, beginning from total records.

## Abbreviations

US: United States
ICD: International classification of disease
PICO: Program-induced cumulative overload
